# Six Sigma in Health Literature, What Matters?

**DOI:** 10.3390/ijerph18168795

**Published:** 2021-08-20

**Authors:** Ana-Beatriz Hernández-Lara, Maria-Victoria Sánchez-Rebull, Angels Niñerola

**Affiliations:** Department of Business Management, Faculty of Business and Economics, University Rovira i Virgili, 43204 Reus, Spain; anabeatriz.hernandez@urv.cat (A.-B.H.-L.); angels.ninerola@urv.cat (A.N.)

**Keywords:** six sigma, lean six sigma, meta-analysis, healthcare, health, academic impact, altmetrics

## Abstract

Six Sigma has been widely used in the health field for process or quality improvement, constituting a quite profusely investigated topic. This paper aims at exploring why some studies have more academic and societal impact, attracting more attention from academics and health professionals. Academic and societal impact was addressed using traditional academic metrics and alternative metrics, often known as altmetrics. We conducted a systematic search following the PRISMA statement through three well-known databases, and identified 212 papers published during 1998–2019. We conducted zero-inflated negative binomial regressions to explore the influence of bibliometric and content determinants on traditional academic and alternative metrics. We observe that the factors influencing alternative metrics are more varied and difficult to apprehend than those explaining traditional impact metrics. We also conclude that, independently of how the impact is measured, the paper’s content, rather than bibliometric characteristics, better explains its impact. In the specific case of research on Six Sigma applied to health, the papers with more impact address process improvement focusing on time and waste reduction. This study sheds light on the aspects that better explain publications’ impact in the field of Six Sigma application in health, either from an academic or a societal point of view.

## 1. Introduction

Six Sigma seeks quality, understood as less variability in a process result [1]. Despite it coming from the manufacturing industry, as Motorola created it, it has been applied to a diverse set of non-manufacturing-related issues, giving excellent results [2,3]. It is based on the principle of measuring, monitoring, and controlling processes through the DMAIC steps (define, measure, analyze, improve, and control) [4]. The goal is to reach 99.9997% accuracy, with only 3.4 defects per million opportunities.

This management philosophy is widely used in the health sector to reduce error, cost, and time [5,6,7,8]. In today’s complex environment, with financial constraints on the healthcare system, increased efficiency could help health institutions to maintain or improve outcomes [9]. In this sense, the use of Six Sigma for addressing health process improvements is gaining scholars’ and professionals’ interest [10]. Most of this research is carried out through case studies showing Six Sigma implementations in different areas of a medical organization [5].

As the field attracts more attention, the impact of its publications also grows. In this regard, citations have been the traditional way to assess research impact [11,12], as well as journals’ impact factor [13], or other indexes, such as the FWCI (Field-Weighted Citation Impact). FWCI is an indicator that compares the actual number of citations received by a document with the expected number of citations for documents of the same type (article, review, book, or conference proceeding), publication year, and subject area [14]. The relevance of these traditional metrics is based on their use in performing evaluations of individual academics, research groups, and universities [15]. However, the criticism received for these classic metrics approaches is growing because they do not analyze the reasons for citations or the impact that research exerts beyond academia [16]. Besides, in the specific case of the health field, the time normally required to accumulate citations may overlook important societal and clinical impacts and new scholarly channels are increasingly used to disseminate scientific results [16].

This critical movement has led to the rise of alternative metrics, commonly known as altmetrics, representing another way of assessing research impact based on relationships and sharing academic publications in online environments [17]. They capture the relevance of research based on metrics such as article views, downloads, and mentions on social media or news media [16], considering channels such as Twitter, Mendeley, CitedULike or blogging [18,19], among others. Given the growth and relevance of social media for sharing scientific knowledge [20], these novel metrics have become necessary and represent another way to assess research impact.

Altmetrics complement citation analysis and other traditional metrics; they overcome their limitations and provide new insights into research impact study [16]. At the same time, altmetrics can provide better signaling of significant publications according to different audiences, which do not necessarily need to be academic, being more suitable for capturing the societal impact of research [21].

Previous research on traditional and altmetric scores of publications has mostly been interested in analyzing the relationship between both kinds of metrics [22]. The conclusions of these studies are not unanimous. While some of them find some degree of correlation between altmetrics and citations [22], others claim that altmetrics cannot predict future citations [23] and that some altmetrics are only weakly correlated with traditional citation metrics [24]. These conflicting results suggest that these two approaches are related; they complement each other, but they do not provide identical information on the visibility and impact of academic research. The conflicting results also point out the necessity of conducting more studies providing additional empirical evidence to support the notion of the determinants that explain what is important in order to increase the citations and the altmetric scores.

In the health field, and more specifically among the extensive literature that considers Six Sigma for addressing health process improvements, we are unaware of any study that has analyzed the determinants that have more impact in the citations and altmetric scores obtained by these publications.

The paper aims to determine which factors are the determinants of the impact of the publications, considering traditional and alternative impact indicators. Specifically, we aim to do this in health publications applying Six Sigma.

These different ways of addressing impact represent important opportunities for academics that concern the objectives of their research. Academics want their contributions to reach the greatest audience. Therefore, they will value finding out the main determinants of their research impact.

After explaining the methodology in Section 2, results are presented in Section 3, and finally, discussion and conclusions are summarized in Section 4 and Section 5, respectively.

## 2. Method

### 2.1. Search Strategy

A systematic search following the PRISMA (Preferred Reporting Items for Systematic Reviews and Meta-Analyses) statement was carried out [25]. We used three databases for guaranteeing greater coverage [26]: WoS (Web of Science) core collection, Scopus, and Medline, for its relevance at the medical level. The analysis included research articles [27] published until 2019, written in English and gathered through the next search engine:

- 1st level (terms related to the methodology should appear in the title): “six sigma” or “six-sigma” or DMAIC;
- 2nd level (terms related to quality processes should be in the Topic for WoS and Medline or in TITLE-ABS-KW for Scopus): “quality systems”, “quality improvement”, or “quality management”;
- 3rd level (the activity sector should be in the Topic for WoS and Medline or in TITLE-ABS-KW for Scopus): “health *”.

**Figure 1:**
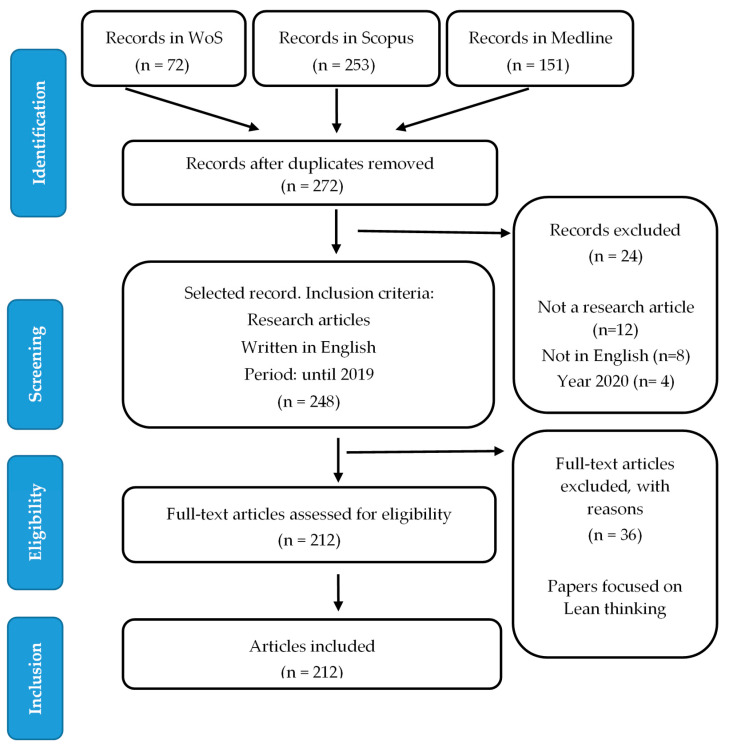
PRISMA.

Figure 1 shows the process carried out for reaching the final sample analyzed, 212 articles.

### 2.2. Variables Examined

#### 2.2.1. Dependent Variable

The impact of the publications can be measured with different indicators, typically separated into traditional or alternatives. Previous research claims they have a different nature, related to academic or social parameters [12]. While traditional metrics assess the impact of a publication based on citations, altmetrics are based on social networks.

Among the traditional metrics, we choose the citations (citations per year to diminish the influence of tenure) and FWCI in the study, as they are commonly accepted in academia [14].

Mendeley and the databases consulted provide much data used as alternative metrics for assessing impact to capture the research’s societal impact. In this sense, we had information on paper usage regarding abstract views, full-text views, link-outs, or times downloaded, besides mentions on Twitter or readers on Mendeley [28].

In the analysis, we included Mendeley readers and the abstract views. Most of the papers had information related to those variables. On the other hand, only 46 papers of the sample showed interaction on Twitter, causing us to choose another alternative social evaluation tool such as Mendeley. Moreover, previous studies claim that an estimated one-third of the scientific community comprises readers [16]. Therefore, the selected variables can gather the scope that research can obtain.

In Table 1, descriptive data of those dependent variables are detailed. As can be seen, except for abstract views, the information is available for all the sample papers. Abstract views also have the highest mean and greater dispersion.

#### 2.2.2. Independent Variables

The independent variables were the determinants that may influence the research impact. These determinants are varied. Research has pointed out that bibliometric information can make the paper more remarkable, easy to find, or more visible for an interested audience [29]. Besides, the content addressed by the research, including their objectives, themes, or the methodologies applied, can also serve as an attractor for gaining readers [30].

Among the bibliometric predictors, previous literature identifies the authors and their bibliometric characteristics as variables influencing the publication impact [12,31], e.g., recognized authors or top authors with many papers and citations typically gather more attention [32], as well as authors from leading universities [33].

We considered several items regarding authorship, such as the number of authors of the paper (N_authors), the type of authorship (academic/professional/both), and the first author’s information, as a proxy of authors’ impact [34]. In this sense, we included the URC (university of affiliation research score) of the first author according to the *Times Higher Education* ranking [35], first author citations the year before the publication of the paper for capturing its notoriety, and the total number of documents he/she authored (first author citations, first author N_papers).

We also included bibliometric variables regarding the source of the publications [36]. It is expected that well-known journals positively contribute to the impact of the research. This means considering the impact factor, the number of fields or categories indexed, or its quartile for addressing journal influence on a paper’s impact. The quartile is used to evaluate the relative importance of a journal within the total number of journals in its category. Therefore, if we divide a list of journals ordered from highest to lowest impact index into four equal parts, each of these parts will be a quartile. We chose Scopus metrics as most of the papers were indexed in this database (SJR, Q, and N_fields). Previous research found a concordance between JCR and SJR metrics in percentiles and ranks [37]; therefore, any could be considered.

Regarding information extracted from the paper itself [31], we added, on the one hand, bibliometric variables such as tenure, references, and keywords (paper tenure, N_references, and N_keywords) [12,30]. Previous research found that bibliometric characteristics affect the impact of research in terms of citations; its effect on social impact should be analyzed as well.

Among the content determinants of the research impact, we included the paper’s objectives, its main themes, and the unit of analysis contemplated in the research. This means, in our specific case, the health department where Six Sigma was applied. In this sense, based on a recent review [5], the main objectives of using Six Sigma in healthcare are reducing cost, time, waste, and errors (OBJ). Besides, the main themes of the publications were determined through their keywords. To do so, the 10 most recurrent keywords were identified (K_). Finally, Six Sigma has been applied to a great range of hospital departments, including cardiology, laboratory, management, medication & pharmacy, nursing, obstetric, pediatric, radiology, rehab, surgery and anesthesiology, traumatology, and UCI and Emergency.

**Figure 2:**
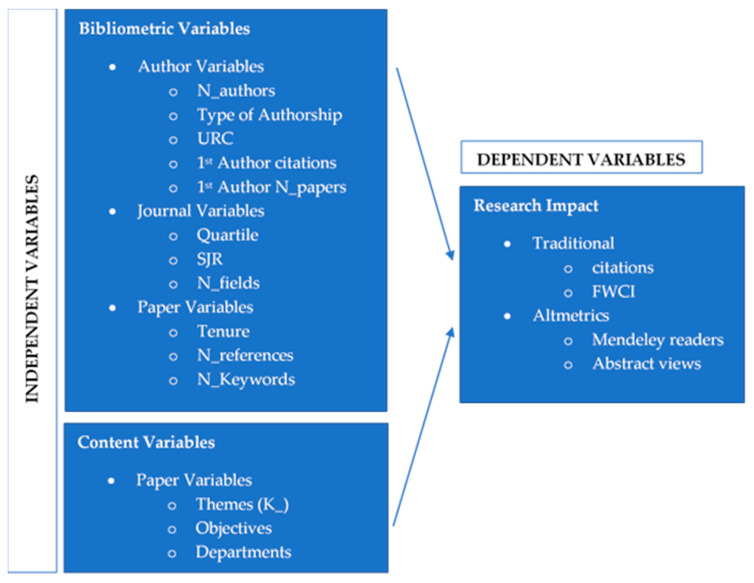
Graphic model of variables analyzed.

Variables included in the model are summarized in Figure 2.

## 3. Results

Statistical data analysis was conducted using RStudio (version 1.4.1103) (RStudio, Inc., Boston, MA, USA) [38]. Firstly, we developed descriptive statistics on the numeric variables and the correlation matrix. The results are shown in Table 1. It shows that the dependent variables were sometimes significantly correlated, but we considered separated regression models for each one. Between the independent variables of the models, the significant correlations only reached moderate levels, confirming that there were no multicollinearity problems.

The results of the Shapiro–Wilk normality test revealed that dependent variables did not follow a normal distribution. Moreover, there was a preponderance of zeros and highly dispersed data, with variances much higher than the means of the dependent variables, as Table 1 shows. Therefore, we used zero-inflated negative binomial regressions for the model estimation.

Table 2 shows the estimation models for the traditional impact metrics (Models 1 refers to citations per year as the dependent variable, and Models 2 has FWCI as the dependent variable). Table 3 shows the estimation models for altmetrics (Model 3 refers to Mendeley readers and Model 4 to abstract views as dependent variables, respectively). Each of the four models was split in two, labeled as submodel a (including the bibliometric determinants) and b (the paper’s content variables).

Overall, all models had excellent goodness of fit (*p* < 0.001) considering the likelihood-ratio tests and Wald tests. It is noteworthy that the AIC (Akaike information criterion) is always lower for estimation models based on the publications’ content than for the models based on the bibliometric indicators. This means that the content models exhibit a better fit, and therefore, the content indicators better explain the impact of the publications, with independence for how this impact is measured.

### 3.1. Traditional Metrics Models

Focusing on the bibliometric models’ results (models 1a, 2a), they show a significant and negative influence of the lower quartiles (Q3 and Q4) on all impact metrics, especially on traditional metrics. On the other hand, we found that publishing in Q2 is not significantly worse than publishing in Q1.

Particularly for citations, even if they are corrected using the citations per year, tenure appears significant and positive (β = 0.038, *p* < 0.05), as well as the number of papers authored by the first author (β = 0.030, *p* < 0.001). Old articles written by a productive principal author achieve greater success in terms of citations. Besides, the number of references seems to affect both traditional metrics (β = 0.014, *p* < 0.001 (citations), β = 0.007, *p* < 0.1 (FCWI)), which means that the use of more references impacts positively on citations.

Moreover, when we consider the impact of content indicators on traditional metrics, it is noteworthy that some keywords positively affect citations and FWCI. We can conclude from the content models that papers using healthcare, process improvement, and quality improvement as keywords have a significantly higher impact in terms of citations and FWCI. In particular, articles addressing process improvement are highly cited (β = 1.384, *p* < 0.01 (citations), β = 1.827, *p* < 0.001 (FCWI)). Additionally, some objectives have more interest for academics considering the traditional metrics. Papers pursuing time and waste reduction are more significantly cited and have higher FWCI according to our results (β = 0.917, *p* < 0.001 and β = 1.478, *p* < 0.001 (citations), β = 0.743, *p* < 0.05 and β = 1.312, *p* < 0.05 (FCWI)).

We could not find any significance regarding the influence of the health departments on FWCI. However, the results show a slightly significant and negative relationship between nursing (β = −1.146, *p* < 0.1) and pediatrics (β = −2.902, *p* < 0.05) compared to cardiology, which was the default department category in the models, concerning the citations.

### 3.2. Altmetrics Models

In the estimation models for the altmetric scores, we found some conflicting results compared with traditional metrics, and less consistency between the effects of the variables analyzed in both dependent variables (Mendeley readers and abstract views). Despite the explanatory capacity of the models, there is much information included in the intercept. This did not occur in the traditional models, giving us insights into the variety of variables that could affect altmetrics.

Models 3a and 4a on bibliometric indicators show the same significant and negative influence of the lower quartiles (Q3 and Q4) also found in traditional metrics. Moreover, tenure plays a negative role in Mendeley readers and abstract views (β = −0.074, *p* < 0.001 and β = −0.072, *p* < 0.05, respectively). Contrary to what occurred with citations and FWCI, new papers achieve more social impact. This result is explained as the most recent articles are usually more promoted on social networks or appear in the researchers’ alerts when new works related to their topic are published.

In those models also, the number of keywords was a significant and positive variable. Using more keywords positively affects both metrics (β = 0.079, *p* < 0.01 (Mendeley readers), β = 0.139, *p* < 0.01 (abstract views)). On the contrary, papers included in more categories, i.e., less focused on a specific research area, experienced a negative relationship with altmetric scores (β = −0.17, *p* < 0.01 (Mendeley Readers), β = −0.265, *p* < 0.05 (abstract views)). Finally, there are two variables with significant impact on abstract views: the number of authors, with a positive influence (β = 0.130, *p* < 0.01), and the SJR that appears to have a negative impact (β = −0.879, *p* < 0.01). This last finding is quite unexpected and is evidence that the scholars find and consider abstracts interesting for themselves, even if they do not belong to high-ranked journals. However, these results are only proven on a specific altmetric score, so more empirical evidence would be necessary to obtain robust conclusions.

The content models, model 3b and 4b results, showed that the papers addressing time reduction are the most relevant for causing researchers to view the papers’ abstract (β = 1.336, *p* < 0.01), while for Mendeley readers, only error reduction is an objective without impact; Mendeley readers are significantly interested in the rest of the identified objectives of this research.

There are several keywords with a significant and positive influence on content scores. The most influential are process improvement (β = 1.405, *p* < 0.01 for readers and β = 3.349, *p* < 0.001 for abstract views), quality improvement (β = 0.853, *p* < 0.001 for readers and β = 1.684, *p* < 0.001 for abstract views), and quality management (β = 2.990, *p* < 0.001 for abstract views). These findings agree with those obtained for traditional metrics where process and quality improvement were also the most influential keywords. Additionally, keywords such as Lean Six Sigma or healthcare also exerted a significant and positive influence on bibliometrics, while Six Sigma or just Lean yielded a negative impact on some of the altmetrics considered.

Finally, our results point out that the department investigated in the publications does not seem to explain their impacts in terms of readers. On the contrary, it is more important to explain the abstract views, especially in publications on rehab. Some others were also significant but negative in comparison with cardiology, which is the default category department. This is the case for obstetric, trauma, or management departments.

## 4. Discussion

Research impact can be addressed using different metrics and can also be affected by various determinants. Although some previous research found a positive correlation between traditional metrics and altmetrics [20,39], others claim they are weakly correlated [24]. Our study has also constated that the items influencing both metrics are different [29]. However, in both cases, the content better explains the impact of the publications rather than bibliometrics.

We have concluded that the determinants affecting traditional metrics are clearer and more specific, so researchers know better what is important to consider when they address improving the impact of their publications in terms of traditional scores based on citations. In this sense, papers published in lower-ranked journals (journals in the Q3 and Q4 of their database) show lower values in traditional metrics. On the contrary, including more references positively affects traditional metrics. Citing other works is a way of gaining visibility. Authors usually receive alerts when they are mentioned, and they may be tempted to read or share the study where it is cited.

The number of previous papers of the first authors seems to be only important when we look at the citations. This finding has a logical explanation. First of all, citations do not exclude self-citations, so authors with previous papers can increase their citations, including their own works. Moreover, if an author is working in the field or has some research experience, his/her work can be better known, compared to novel authors. This fact does not occur in the other traditional metric considered. FWCI compares the actual number of citations received by a document with the expected number of citations based on some aspects. So, the power or influence of an experienced first author may have less weight.

Altmetrics, on the contrary, more based on the societal impact of research, can be influenced by varied determinants beyond bibliometric indicators, especially indicators related to the content of the publications. A paper may have more readers or views because of other reasons, that countenance moving beyond the traditional standards of academia [40]. Baek et al. [41] analyzed the top-cited articles versus top altmetric articles in a particular field, finding no overlaps between the two samples. This confirms that traditional and academic metrics do not always go in the same direction [40].

Previous research agreed that journals’ impact is one of the most relevant determinants of citations and altmetrics [29]. In this study, we emphasize the importance of the quartile where the journal is positioned more than its impact factor. As a matter of fact, our results point out that abstract views can be higher in the case of journals not well ranked, perhaps because these journals make more effort to attract a bigger audience, knowing that visualization is the first step to attract future readers and higher impact. Moreover, other article characteristics such as authorship or the number of references increase the article’s impact, especially in traditional metrics [29,31]. Considering the bibliometric determinants of impact, it is also noteworthy to highlight the different effects of tenure. We agree with previous research that altmetrics can provide more real-time information [13], as the effect of tenure works contrarily than for traditional metrics. Similar to Araújo et al. [40], our results also confirm that recent publications receive more attention with altmetrics, and older, seminal works benefit from using more conventional metrics for measuring research impact.

As aforementioned, we found that the content models better explain the data. This means that papers analyzing Six Sigma in the health field are more cited or have higher social visibility for what they investigate (their main objectives, themes, and the units of analysis) rather than for their bibliometric characteristics. Those results align with other previous research that pointed out the research topic as the key to success [42]. According to our results, the objective of the article is essential for achieving academic and societal impact. In this regard, time and waste reduction are the main goals of highly cited papers in the studied field. Moreover, the more relevant themes were common for traditional and altmetric scores, which were related to process and quality improvement and quality management. The relevance of the objective analyzed and the theme addressed by the publication was higher than the impact of the unit of analysis or the department considered in the research.

Table 4 summarizes the main effects found, highlighting the importance of the content of the paper in all metrics. In red are the determinants that negatively affect the impact of the publication; in green, the positive.

## 5. Conclusions

This research provides additional evidence on the determinants that explain what matters in order to increase the scientific and societal impact of research on Six Sigma applied to health. There is an open debate on the scope and determinants that influence the different metrics that capture the impact of publications. Moreover, besides providing more evidence to support the inconsistent findings of previous research on the topic, we also pointed out the relevance of this kind of study in the health field, because in this case, the time required for traditional metrics of impact may overlook important scientific advances with societal and clinical impacts, which justifies the relevance of alternative metrics in this field and the need to understand their determinants better [18].

As a conclusion, our results confirm that it is more complicated to apprehend the varied factors that explain the societal impact of academic research. Contrary to traditional metrics, which are more stable, influenced by time, and solidly based on bibliometric and content determinants, altmetrics can also be affected by other factors. They are not as well known, given the novelty of social media and academic networks to divulge research, and also given the varied methods and tools that journals and scholars themselves can use to promote and obtain higher visibility.

Despite these difficulties, there are some determinants in the bottom-line of impact, independently of how it is measured, which allow us to reach conclusions on the relevance of the paper’s content rather than their bibliometric characteristics.

In the specific case of research on Six Sigma applied to health, process, and quality improvement, the themes and objectives that assure the highest impact, for both traditional and alternative metrics, are addressed mainly by time and waste reduction, independently of the department or unit of analysis used.

However, even if this kind of study provides additional insights into what matters to enhance the academic and societal impact of research, it is necessary to mention the limitations in obtaining these alternative metrics. Data quality problems are usual and constitute a relevant issue in the field, inviting us to consider the findings with caution and suggesting the need to make additional efforts in the future to overcome this limitation.

Although it seems complicated that citations and the journal impact factor stop being crucial, currently the use of social networks and support software, like Mendeley, are enhancing the relevance and weight of altmetrics in academic research.

Despite altmetrics not representing an alternative to the traditional methods to measure research output impact, some organizations such as DORA (Declaration on Research Assessment) [43] are pressing to improve how scientific research is evaluated by funding agencies, academic institutions, and other parties, trying to go beyond traditional indicators such as the impact factor of the journal.

In this sense, Scopus has a tool known as PlumX Metrics [44] that gathers people’s footprints when interacting with research and categorizes them into five categories—Usage, Captures, Mentions, Social Media, and Citations. It would be interesting for all parties involved in research to establish an overall indicator giving different weights to those categories to find a representative parameter agglutinating all the impact indicators. Thus, both metrics, academic and societal, could contribute to assessing the impact of a publication.

## Figures and Tables

**Table 1:** Descriptive data and correlation matrix of the numeric variables.

	N	Mean	sd	1	2	3	4	5	6	7	8	9	10	11	12	13
1. Citations per year (c/y)	212	2.42	3.18	1												
2. FWCI	212	1.52	1.90	0.570 ***	1											
3. Mendeley readers	212	16.46	24.60	0.581 ***	0.607 ***	1										
4. Abstract views	157	76.30	131.47	0.215 *	0.508 ***	0.524 ***	1									
5. URS	76	46.39	23.61	0.128	−0.199	−0.141	−0.165	1								
6. N_Authors	212	3.68	2.80	−0.327 *	−0.371	−0.458 **	−0.308	0.182	1							
7. 1st author citations	212	217.36	772.18	−0.027	−0.108	−0.198 **	−0.292	−0.131	−0.078 **	1						
8. 1st author N_papers	212	7.95	9.95	0.158 **	−0.121 *	−0.198 **	−0.157	−0.076	−0.057 **	0.589 ***	1					
9. N_fields	212	2.15	1.07	−0.336	−0.505	−0.536	−0.554 *	0.121 *	0.309	0.023	0.312	1				
10. SJR	212	0.61	0.55	−0.342 **	−0.287 *	−0.188 **	0.159	−0.377	0.119 ***	0.154 **	−0.154 **	−0.185	1			
11. Paper tenure	212	8.36	5.41	0.178	−0.132	−0.302 ***	−0.336 **	−0.009 ***	0.116 ***	0.168	0.409	0.190	0.081 ***	1		
12. N_references	159	28.52	22.17	0.427 ***	−0.015	0.397 **	0.185	−0.176	−0.101	−0.036	0.078	0.027	−0.065	−0.186 *	1	
13. N_keywords	212	2.94	2.76	0.369 ***	0.260	0.215 ***	0.262 *	0.012	−0.202 *	0.181	0.375	0.146 ***	−0.239	−0.076 ***	0.576 *	1

Correlation significant at the level of * 0.05, ** 0.01, and *** 0.001 (bilateral).

**Table 2:** Zero-inflated negative binomial regression model for traditional metrics of research impact.

	Citations Per Year				FWCI			
	Model 1a: Bibliometric		Model 1b: Content		Model 2a: Bibliometric		Model 2b: Content	
	EST	SE	EST	SE	EST	SE	EST	SE
Intercept	0.157	0.366	−0.346	0.527	0.732	0.418 +	−0.587	0.623
N_Authors	0.028	0.024			−0.003	0.029		
N_Keywords	0.049	0.029			0.017	0.034		
Author_Prof	0.383	0.198 +			0.348	0.227		
Author_Both	−0.125	0.163			−0.033	0.190		
1st author citations	−0.001	0.001			0.000	0.000		
1st author N_papers	0.030	0.009 **			0.008	0.012		
N_Fields	−0.057	0.068			−0.120	0.080		
Q2	−0.273	0.183			−0.323	0.208		
Q3	−1.821	0.342 ***			−2.263	0.450 ***		
Q4	−2.026	0.614 ***			−2.261	0.802 **		
SJR	−0.144	0.167			−0.308	0.201		
Paper tenure	0.038	0.015 *			0.027	0.017		
N_references	0.014	0.003 ***			0.007	0.004 +		
OBJcost			0.379	0.241			0.149	0.292
OBJtime			0.917	0.269 ***			0.743	0.327 *
OBJwaste			1.478	0.432 ***			1.312	0.521 *
OBJerror			0.256	0.229			0.184	0.280
Laboratory			−0.080	0.465			−0.568	0.590
Management			−0.251	0.438			−0.605	0.512
Med&Pharma			0.108	0.570			0.060	0.658
Nursing			−1.146	0.608 +			−0.635	0.605
Obstetric			−0.670	0.685			−1.008	0.847
Pediatric			−2.902	1.272 *			−2.627	1.390
Radiology			−0.153	0.480			0.339	0.512
Rehab			0.838	0.846			0.699	1.055
Surgery&Anesthesiology			0.206	0.402			0.059	0.462
Trauma			0.560	0.414			0.057	0.491
UCI&Emergency			−0.640	0.457			−0.520	0.518
K_DMAIC			0.362	0.399			−0.059	0.516
K_Healthcare			0.635	0.254 *			0.667	0.299 *
K_Hospital			−0.063	0.426			−0.350	0.513
K_Lean			0.249	0.308			0.252	0.356
K_LSS			0.309	0.257			0.543	0.325 +
K_Process improvement			1.384	0.455 **			1.827	0.507 ***
K_Quality improvement			0.510	0.234 *			0.785	0.283 **
K_Quality management			0.516	0.341			0.440	0.435
K_SS			−0.110	0.224			−0.198	0.272
K_Waiting time			−0.049	0.307			0.086	0.396
AIC	611.88		302.72		516.15		261.33	
Log-likelihood	−290.94 (df = 15)		−124.36 (df = 27)		−243.075 (df = 15)		−103.67 (df = 27)	
Lrtest null. Model (Chi squared)	100.49 ***		70.087 ***		63.319 ***		58.399 ***	
Wald test (F)	7.509 ***		6.299 ***		3.521 ***		3.428 ***	

Significant at the level of + 0.10, * 0.05, ** 0.01, and *** 0.001 (bilateral). Note: EST: estimate; SE: standard error. 1st author: first author information.

**Table 3:** Zero-inflated negative binomial regression model for alternative metrics of research impact.

	Mendeley Readers				Abstract Views			
	Model 3a: Bibliometric		Model 3b: Content		Model 4a: Bibliometric		Model 4b: Content	
	EST	SE	EST	SE	EST	SE	EST	SE
Intercept	2.841	0.340 ***	1.230	0.581 *	5.410	0.665 ***	2.092	0.926 *
N_Authors	−0.006	0.025			0.130	0.047 **		
N_Keywords	0.079	0.028 **			0.139	0.053 **		
Author_Prof	0.005	0.192			−0.213	0.375		
Author_Both	0.189	0.149			−0.381	0.291		
1st author citations	0.000	0.000			−0.0001	0.0002		
1st author N_papers	0.013	0.010			0.006	0.019		
N_Fields	−0.170	0.063 **			−0.265	0.125 *		
Q2	0.163	0.180			−0.598	0.348 +		
Q3	−0.819	0.119 ***			−0.422	0.460		
Q4	−0.962	0.352 **			−1.691	0.676 *		
SJR	0.145	0.165			−0.879	0.326 **		
Paper tenure	−0.074	0.015 ***			−0.072	0.031 *		
N_references	0.011	0.003 ***			−0.007	0.006		
OBJcost			0.777	0.316 *			0.537	0.488
OBJtime			0.629	0.293 *			1.336	0.458 **
OBJwaste			1.795	0.649 **			−1.169	1.014
OBJerror			0.324	0.258			0.051	0.397
Laboratory			0.699	0.505			−0.075	0.729
Management			0.729	0.502			−2.674	0.758 ***
Med&Pharma			0.259	0.631			−0.958	0.960
Nursing			0.077	0.549			0.320	0.755
Obstetric			0.850	0.756			−2.598	1.087 *
Pediatric			−1.730	1.076			−2.195	1.602
Radiology			0.431	0.550			−0.899	0.858
Rehab			1.070	1.022			3.298	1.460 *
Surgery&Anesthesiology			0.414	0.482			−0.049	0.709
Trauma			0.381	0.532			−1.599	0.752 *
UCI&Emergency			0.469	0.508			−1.215	0.739 +
K_DMAIC			0.268	0.457			0.092	0.692
K_Healthcare			0.573	0.297 +			2.084	0.462 ***
K_Hospital			−0.558	0.475			1.527	0.797 +
K_Lean			0.200	0.359			−1.338	0.546 *
K_LSS			0.684	0.285 *			2.109	0.510 ***
K_Process improvement			1.405	0.583 **			3.349	0.963 ***
K_Quality improvement			0.853	0.243 ***			1.684	0.420 ***
K_Quality management			−0.005	0.360			2.990	0.712 ***
K_SS			−0.507	0.247 *			0.185	0.464
K_Waiting time			0.022	0.389			−0.123	0.610
AIC	1179		625.23		1385		700.36	
Log-likelihood	−574.51 (df = 15)		−285.62 (df = 27)		−677.5 (df = 15)		−323.18 (df = 27)	
Lrtest null. Model (Chi squared)	112.22 ***		66.136 ***		32.128 **		52.338 **	
Wald test (F)	11.51 ***		3.289 ***		3.2318 ***		6.063 ***	

Significant at the level of + 0.10, * 0.05, ** 0.01, and *** 0.001 (bilateral). Note: EST: estimate; SE: standard error. 1st author: first author information.

**Table 4:** Most important research impact determinants.

	Citations Per Year	FWCI	Mendeley Readers	Abstract Views
1st author N_papers	positive			
N_Fields			negative	negative
Q3	**negative**	**negative**	negative	
Q4	**negative**	**negative**	negative	**negative**
Paper tenure	positive		negative	negative
N_references	positive	positive	positive	
OBJtime	positive	positive	positive	**positive**
OBJwaste	**positive**	**positive**	**positive**	
K_Healthcare	positive	positive	positive	**positive**
K_Process improvement	**positive**	**positive**	**positive**	**positive**
K_Quality improvement	positive	positive	positive	**positive**

Beta higher than 1 in bold. Green: positive impact. Red negative impact.

## Data Availability

Data are available from the authors upon reasonable request.
